# Case Report: BCMA-targeting CAR T-cell therapy induces complete and durable remission in relapsed extramedullary plasmablastic multiple myeloma

**DOI:** 10.3389/fimmu.2025.1567403

**Published:** 2025-05-26

**Authors:** Lili Zhou, Paul Ning Man Cheng, Jian-qing Mi

**Affiliations:** ^1^ Department of Hematology, Shanghai Sino United Hospital, Shanghai, China; ^2^ Department of Medicine, The Chinese University of Hong Kong (CUHK), Hong Kong, Hong Kong SAR, China; ^3^ State Key Laboratory of Medical Genomics, National Research Center for Translational Medicine, Shanghai Institute of Hematology, Ruijin Hospital Affiliated with Shanghai Jiao Tong University School of Medicine, Shanghai, China

**Keywords:** BCMA CAR-T, extramedullary, plasmablastic multiple myeloma, Equecabtagene Autoleucel, maintenance

## Abstract

Plasmablastic multiple myeloma (PBM) is an aggressive multiple myeloma (MM) form, identified by a high risk of recurrence and poor prognosis, with limited effective treatment options. Present study reports a case initially diagnosed with IgG-kappa MM with double-hit genetics. Following induction chemotherapy with bortezomib, doxorubicin and dexamethasone (VAD), and subsequent consolidation therapy with ixazomib, lenalidomide, and dexamethasone, the disease progressed, manifesting as a plasmoblastic tumor in the right pelvic cavity. After two cycles of carfezomib, daratumumab, cyclophosphamide, cisplatin, etoposide and dexamethasone (KD-DECP), the patient achieved partial response. She declined autologous stem cell transplantation (ASCT) and instead received radiotherapy as bridging therapy, followed by B-cell maturation antigen (BCMA)-targeting chimeric antigen receptor (CAR) T-cell therapy with pomalidomide as maintenance therapy. She achieved complete response (CR) at 3 months and has remained disease-free for over 15 months based on the latest follow-up. Although grade 2 cytokine release syndrome (CRS) and other adverse events were observed, they were manageable. BCMA CAR-T cell accompanied with bridging radiotherapy and pomalidomide as maintenance therapy provided a promising therapy treatment for PBM, which is more aggressive and with shorter survival. Further studies are demanded to assess the efficiency and long-term benefits for this challenging subtype.

## Introduction

In rare cases, the plasma cells infiltrate other tissues outside the bone marrow (BM) including lymph nodes, soft tissue, the central nervous system, etc., a condition known as extramedullary disease (EMD) (1), which usually correlates to a significantly poorer prognosis compared to those without EMD (2). Plasmablastic multiple myeloma (PBM) accounts for approximately 8.2% all multiple myeloma (MM) cases (3) and is often challenging to distinguish from plasmablastic lymphoma (PBL) (4). PBM represents a highly proliferative disease, characterized by unfavorable clinical features, evaluated proliferation index, a high proportion of plasma cell infiltration in BM, abnormal karyotypes, and del (13q) as seen in karyotyping (5). It is also associated with shorter survival (3, 6), particularly during the first 6 months (6).

The incidence of PBM with EMD is very low, but it tends to be more aggressive and is associated with shorter survival (3, 6). Currently, no consensus exists upon the management of patients diagnosed with this condition, and research on treatment options is limited. This article presents the case of a patient with double-hit refractory/relapsed (R/R) MM, accompanied by an extramedullary plasmoblastic tumor. After undergoing BCMA-targeting CAR-T cell therapy, she achieved a CR and has remained disease-free for an extended period. This case provides valuable insights and highlights BCMA CAR-T cell therapy as a promising treatment option regarding this challenging subtype.

## Case presentation

A 61-year-old female patient having pain in chest, back and ribs, with general good condition and had no positive signs, without abdominal pain, abdominal distension, constipation, diarrhea, fever or pain, but with fatigue, weight loss and anemia. Physical examination of the abdomen reveals a mass with a tough texture and without tenderness. Subsequently, a series of laboratory tests were underwent, which led to a diagnosis of IgG-kappa MM with Durie-Salmon stage IIIB and International Staging System (ISS) stage III in July 2020. Fluorescence *in situ* hybridization (FISH) of BM aspirate was positive for 1q21 gene amplification, IGH gene rearrangement, (t(4;14)) IGH/FGFR3 fusion, and RB1 gene deletion, indicating a double-hit disease according to the Mayo Clinic’s mSMART 3.0 program (6). Chromosome karyotype analysis revealed that 43, X, -X, -6, -10, del(12)(p11.2), -13, der(17)t(1;17)(q21; q25), -20, +mar1, +mar2[2]/46, XX [16].

As the patient refused autologous hematopoietic stem cell transplantation (ASCT), she underwent induction therapy of six cycles bortezomib, doxorubicin, and dexamethasone (VAD), which resulted in complete remission. This was followed by four cycles of ixazomib, lenalidomide and dexamethasone (IRD) as consolidation chemotherapy, which was later switched to lenalidomide and dexamethasone alone, or lenalidomide monotherapy, due to poor tolerance of ixazomib and dexamethasone.

The M protein became positive in May 2023 with 1.5%, and the free light chain ratio progressively increased, suggesting biochemical relapse. The patient then noticed a mass in right lower abdomen. Abdominal computed tomography (CT) showcased a mass in the right pelvic cavity, and PET/CT revealed hypermetabolic uptake in the right lower abdomen with 12.3*6.6*12.9 cm, SUVmax=20.3. Pathological analysis indicated a plasmoblastic tumor, with high proliferative activity and MYC overexpression, both of which are associated with high risk, and a ki-67 index of about 90%. BM aspirate showcased 1% plasma cell infiltration, and following immunophenotypic markers were observed: CD38(bright+), CD56+, CD138+, cKappa+, BCMA(100%)+, CD19-, CD27-, CD28- and cLambda-.

Then, she received two cycles of KD-DECP with halved dosage due to poor tolerance: carfilzomib 20mg/m^2^ on days 1-2, 27mg/m^2^ on days 8-9, 15-16; daratumumab 16 mg/kg weekly, 700 mg on days 1, 8, 15, and 22; cyclophosphamide 400 mg, cisplatin 10 mg, and etoposide 40 mg on days 1-4; and dexamethasone 20 mg on days 1-4, 8-9, and 15-16. During this regimen, the patient developed grade 4 neutropenia and febrile neutropenia. After two cycles of treatment, she was assessed as having a partial response with tumor size 4.0*2.4 cm, but progressed rapidly with free light chain rises after a week.

Given the patient was diagnosed with PBM with high Ki-67 and rapid progression after KD-DECP, which suggested she might not benefit from or tolerate further chemotherapy, and the fact that she refused ASCT, combined with the abundant BCMA expressions on her BM cells and the reported anti-myeloma effects of BCMA CAR-T cell therapy, our group decided to proceed with CAR-T as the next treatment option. Peripheral blood samples were collected to generate the engineered CAR-T cells. The patient underwent radiotherapy for the residual tumor in the abdomen as bridging therapy, with a dose of 10Gy/5 fractions. There was no distinct change in the tumor size after radiotherapy. After 3 days of lymphodepletion with fludarabine and cyclophosphamide, BCMA CAR-T cells (Equecabtagene Autoleucel, Eque-cel) from IASO Biotherapeutics were administered at 1.0×10^6^ CAR-T cells/kg, considering the extremely high risk disease, pomadomide was initiated after 3-month of CAR-T infusion as long-term maintenance therapy.

**Figure 1:**
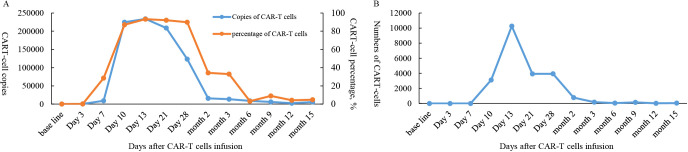
Cell expansion and persistence in peripheral blood following BCMA CAR-T cell infusion. **(A)** Copies and percentage of CAR-T cells. **(B)** Numbers of CAR-T cells.

**Figure 2:**
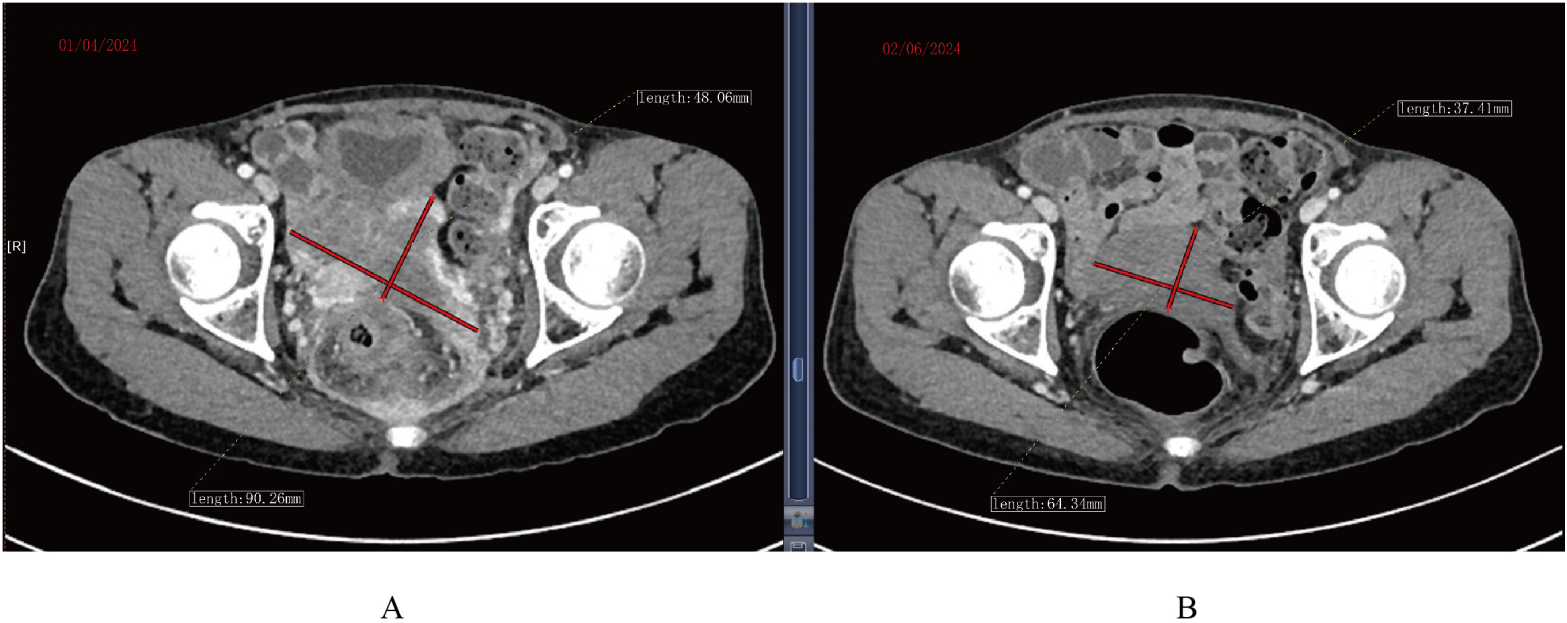
CT scan of tumor mass changes showing partial response 1 month post CAR-T cell infusion. **(A)** CT scan before infusion of CAR-T infusion. **(B)** CT scan 1 month post CAR-T cell infusion.

**Figure 3:**
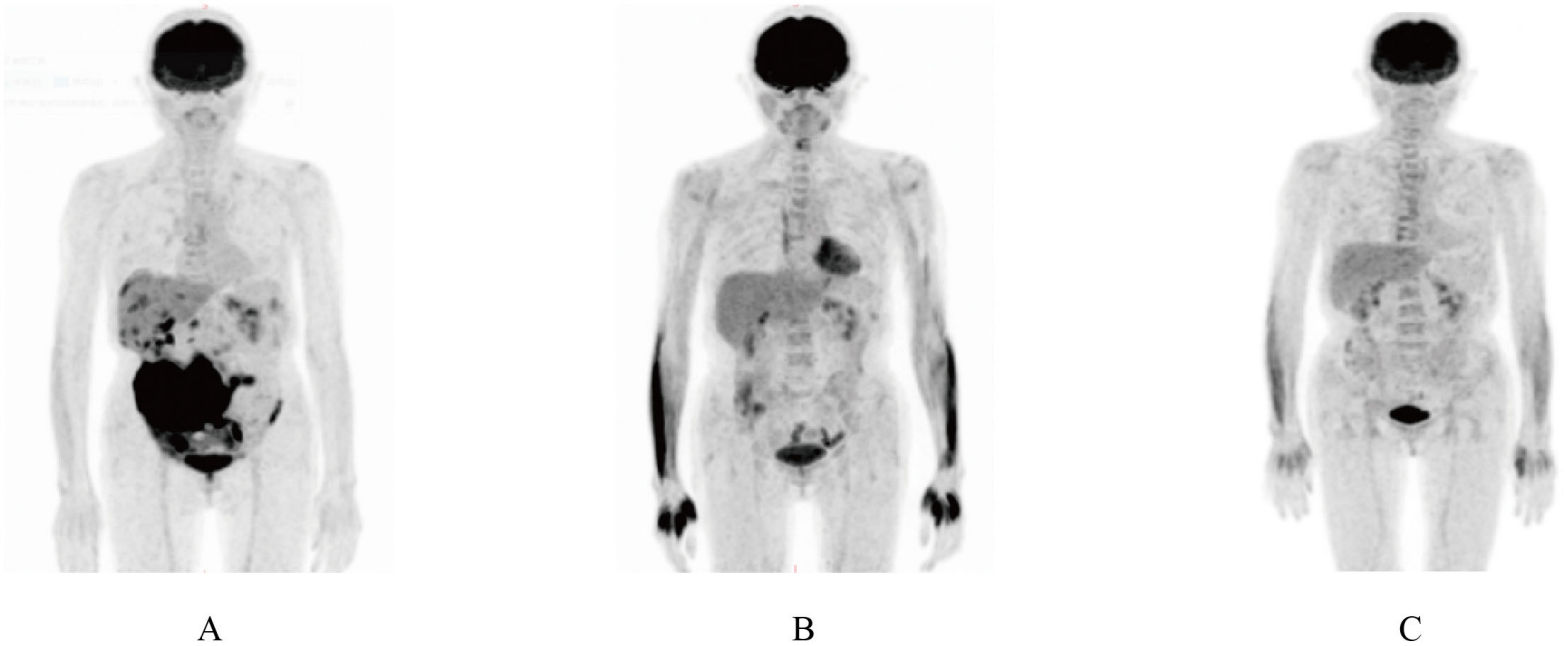
PET/CT scan showing complete remission 3 months after CAR-T therapy. **(A)** PET/CT scan showing a mass in lower right abdomen after induction and consolidation therapy. **(B)** PET/CT scan before CAR-T cell therapy. **(C)** PET/CT scan 3 months after CAR-T cell infusion.

**Figure 4:**
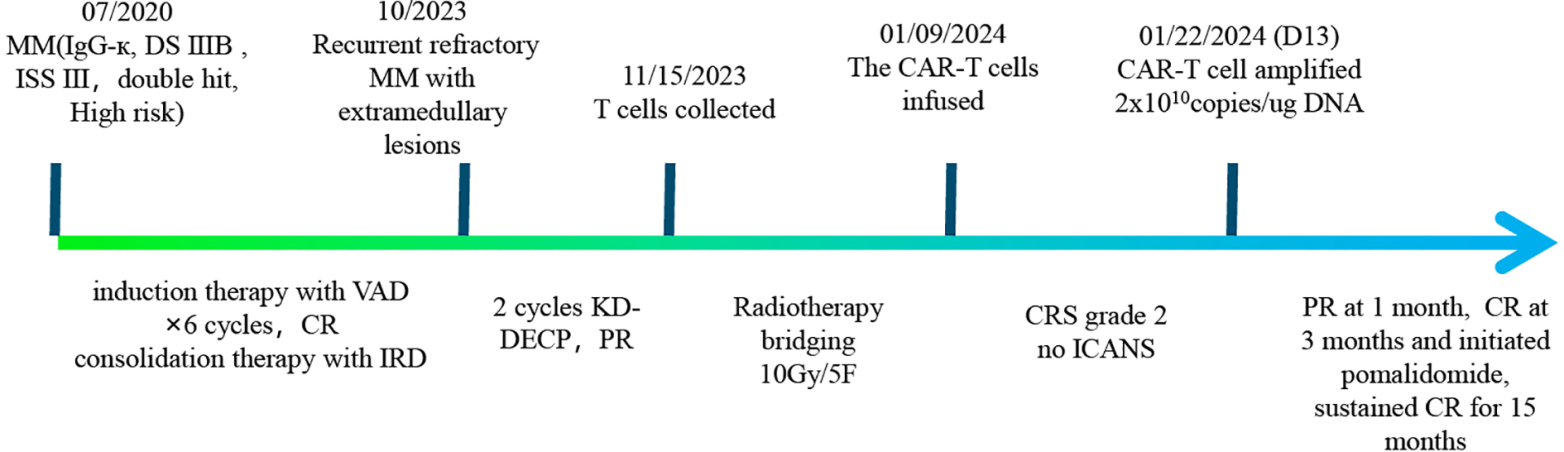
Timeline of patient’s diagnosis and treatment.

Continuous expansion regarding BCMA CAR-T cells was observed, having the peak copy number in peripheral blood reaching 2×10^10^ copies/ug genomic DNA 13 days post-infusion (**Figure 1**). She developed grade II cytokine release syndrome (CRS) for 5 days with IL-6 >5000 pg/ml, IL-8 287.61 pg/ml, IL-10 130.62 pg/ml and IFN-γ517.1 pg/ml, along with neutropenia and thrombocytopenia, which resolved after three months. Two doses of tocilizumab (8mg/kg every 8 hours), steroid 10mg once and ibuprofen were used to manage CRS. No immune effector cell-associated neurotoxicity syndrome (ICANS) happened. She achieved partial response (PR) at 1 month with tumor size 3.7 * 6.4cm (**Figure 2**) and complete remission (CR) at month 3 (**Figure 3**) post CAR-T-cell infusion. Approximately 15 months after CART-cell therapy, she remained sCR (strict complete response) and MRD negative (**Figure 4**).

## Discussion and conclusions

### Discussion

A meta-analysis indicated that high-risk cytogenetic profile significantly correlated to worse overall survival (OS), progression-free survival (PFS), and overall response rate (ORR). Additionally, EMD presence linked to significantly worse OS and PFS comparing to standard-risk genetics (7). The prognosis of PBM was poor than that of other types of MM (6). PBM may appear in BM and/or extramedullary tissues, but PBM with EMD is associated with worse OS than those without extramedullary involvement, with median OS of 17 and 28 months, respectively (8). CAR-T cell therapy may emerge as a potential therapeutic option towards high-risk PBM with EMD. In particular, Eque-cel has shown promising results in MM patients. The FUMANBA-1 trial illustrated that Eque-cel caused durable responses in patients of heavily pretreated refractory/relapsed multiple myeloma (RRMM), with 96.0% ORR and a 12-month PFS rate 78.8% (9). Another study unraveled that the median PFS and OS of Eque-cel in RRMM was 22.6 and 50.2 months, respectively (10). FUMANBA-2 trail showed favorable results, evaluating Eque-cel in newly diagnosed high-risk MM, with a 100% ORR and 71.4% minimal residual disease (MRD) negativity (11).

Currently, BCMA CAR-T is mainly used in patients with RRMM, particularly those with multiple prior lines of therapy, often accompanied by compromised organ function. In this setting, disease progression can be rapid. Following review of PBM data and shared decision-making discussions, the patient demonstrated full comprehension of the poor prognosis, constrained treatment alternatives, and lack of standard therapy for it. Given her documented resistance to and poor tolerance of prior chemotherapy regimens, the patient declined ASCT and elected to proceed with anti-BCMA CAR-T cell therapy.

It has been reported that EMD (12, 13) and high disease burden (14–16) of CAR-T infusion correlate to high risk of higher-grade CRS and ICANS. Bridging therapy is used to control cancer progression as well as reduce the severe CRS and ICANS risks. Amongst potential bridging treatments, radiation therapy (RT) is an efficient one, especially if the tumor is limited. Preclinical evidence demonstrates that RT potentiates CAR-T cell efficacy by increasing tumor antigen exposure, thereby mitigating antigen-negative relapse (17). RT is shown to be effective, which is safe bridging therapy for local cancer control (18–20). A retrospective study reported that patients receiving RT as bridging therapy had significantly better 1-year PFS and OS comparing to patients who received chemotherapy or no bridging therapy. The 1-year PFS was 51.2% in RT group, 28.2% in chemotherapy group, and 47.6% in no-bridging group. The 1-year OS was 86.7%, 52.7% and 69%, respectively (21). Bridging radiotherapy reduces tumor burden in extramedullary multiple myeloma patients without compromising subsequent CAR-T cell infusion, demonstrating significant clinical implications of the interplay between radiation and immunotherapy.

The role of maintenance therapy after CAR T-Cell therapy in relapsed/refractory multiple myeloma (RRMM) remains unclear and investigational. While CAR-T induces deep responses (including MRD negativity in some patients), relapse remains common due to CAR-T cell exhaustion, antigen escape, or tumor microenvironment resistance. Maintenance therapy aims to prolong remission, but standardized guidelines are lacking. Potential Maintenance Strategies including Immunomodulatory Drugs (IMiDs), Anti-CD38 Monoclonal Antibodies (e.g., daratumumab), Anti-BCMA bispecific antibodies (e.g., teclistamab) or ADC (belantamab mafodotin). IMiDs exert multiple anti-MM effects, including anti-angiogenic, anti-proliferative, and immunomodulatory effects, and has been demonstrated to promote T-cell proliferation in an IL-2-dependent manner (22). Investigations have shown that maintenance therapy with pomalidomide and lenalidomide may assist in promoting CAR-T cell re-expansion in patients with high-risk MM (23, 24). Most of the patients who received ciltacabtagene autoleucel lost detectable CAR T cells by 6 months after infusion (25). Maintenance may be initiated after CAR-T recovery (e.g., ≥3 months post-infusion). Overlapping immunosuppression (e.g., infections) must be balanced. BCMA CAR-T cell therapy with long-term pomalidomide resulted in low recurrence rate and manageable adverse effects (24). In patients that had pomalidomide as maintenance therapy post CAR-T cell infusions, median time to progression (TTP) was 13 months, though OS was not achieved. After a median follow-up of 27 months, the median OS and TTP was 10.7 and 5.85 months, respectively, in patients without receiving pomalidomide (24).In this case, considering PBM combined with multiple high-risk factors, we opted for pomalidomide maintenance therapy. At 12 months post-infusion, we detected 2,296 copies of CAR-T cells/μg gDNA. Remarkably, at 15 months, 5,670 copies/μg gDNA were still detectable (**Figure 1**), indicating that the CAR-T persistence duration in this patient has already exceeded the median persistence duration of 419 days reported for Eque-cel (26) The observed increase in expansion during this phase might potentially be attributed to the therapeutic effects of pomalidomide maintenance. We anticipate that this treatment approach may enable the patient to achieve prolonged disease-free survival (DFS).There is currently no established standard maintenance regimen, and participation in clinical trials should be prioritized (e.g., NCT05257083, NCT04832854).Outside trials, individualized decisions based on patient risk, prior therapies, and response depth may include IMiDs, anti-CD38 antibodies, or observation. Long-term data are needed to define optimal strategies.

In conclusion, this case demonstrates that BCMA CAR-T cell therapy, accompanied with RT as bridging therapy and pomalidomide as maintenance therapy, is efficient to treat MM with extramedullary plasmablastic disease, with manageable adverse events. However, further prospective investigations having larger sample sizes are demanded to validate superiority of the therapeutic strategies.

## Data Availability

The original contributions presented in the study are included in the article/supplementary material. Further inquiries can be directed to the corresponding author.
